# Supplementary material for: Replay of large-scale spatio-temporal patterns from waking during subsequent NREM sleep in human cortex
Source: Sci Rep. 2017 Dec 12;7:17380. doi: 10.1038/s41598-017-17469-w (PMC5727134; doi:10.1038/s41598-017-17469-w)
Supplement: Supplementary file 1 — Supplementary Information [file 41598_2017_17469_MOESM1_ESM.pdf]

Title: Replay of large-scale spatio-temporal patterns from waking during subsequent NREM sleep in human cortex

Author affiliation: Xi Jiang[1+]*, Isaac Shamie[1a], Werner Doyle[2], Daniel Friedman[2], Patricia Dugan[2], Orrin Devinsky[2], Emad Eskandar[3], Sydney Cash[4], Thomas Thesen[2], Eric Halgren[1a,5]*

[1] Neurosciences Graduate Program, University of California at San Diego, La Jolla, CA 92093, USA.

[1a] Department of Radiology, University of California at San Diego, La Jolla, CA 92093, USA.

[2] Comprehensive Epilepsy Center, New York University School of Medicine, New York, NY 10016, USA.

[3] Department of Neurosurgery, Epilepsy Division, Massachusetts General Hospital, Harvard Medical School, Boston, MA 02114, USA.

[4] Department of Neurology, Epilepsy Division, Massachusetts General Hospital, Harvard Medical School, Boston, MA 02114, USA.

[5] Department of Neurosciences, University of California at San Diego, La Jolla, CA 92093, USA.

+: Submitting Author

*Corresponding Authors: x4jiang@ucsd.edu (1-845-857-6020), ehalgren@ucsd.edu (1-858-822-8385)

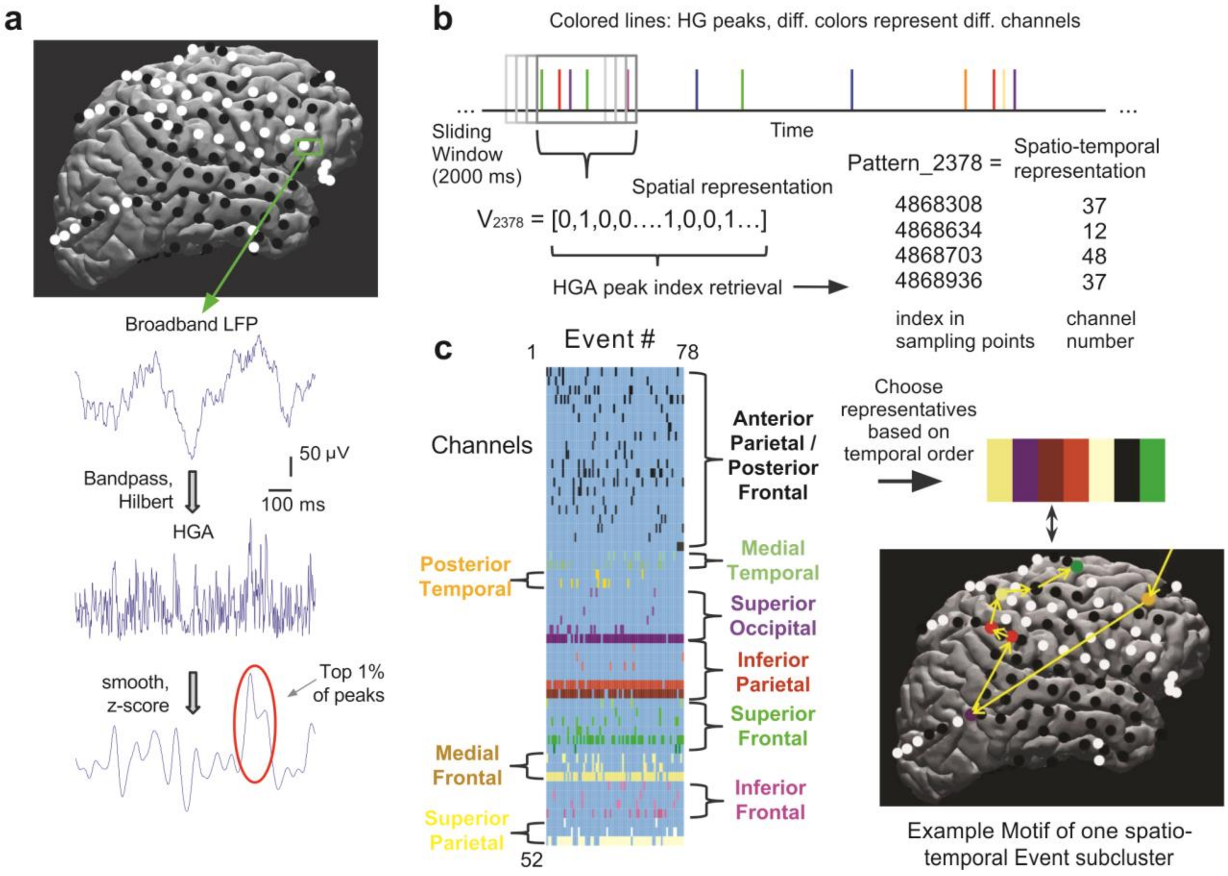

Supplementary Figure S1. Selection of Motifs representing consistent spatiotemporal patterns of HG peaks. ***a***, Selection of HC peaks. Top: ECoG electrode map (right hemisphere) for subject 1, black circles indicating contacts/channels discarded for downstream processing due to interictal activity or, rarely, poor recording quality. Bottom: An example data segment recorded from a grid electrode. The raw time series (LFP) was bandpassed (70-190 Hz), and the Hilbert transform was applied to obtain the HG analytic amplitude. Red circle: a local maximum/peak in HG. Only the top 1 % of all peaks was chosen for each channel as signals of cortical activation. ***b***, Selection of waking activity Events. 2-second sliding windows were moved across time to produce time segments with different numbers of HG peaks (“Events”). The segments that yielded local maxima of HG peak numbers were then transformed into their

corresponding spatial and spatio-temporal representations. ***c***, Clustering of Events into Motifs. The binary representations of waking activity underwent hierarchical clustering based on the spatial information (i.e. channels active). Using the matching index algorithm, Events in each cluster were further divided into subclusters based on the temporal order in which channels produce HG peaks. The representative Events in each subcluster were then chosen as template Motifs, to be matched to the Events found in NREM (N2+N3) periods. In summary, we use "Events" to refer to recurring spatiotemporal patterns of HG peaks, and "Motifs" to refer to clusters of Events with relatively minor differences which are grouped together for analysis.

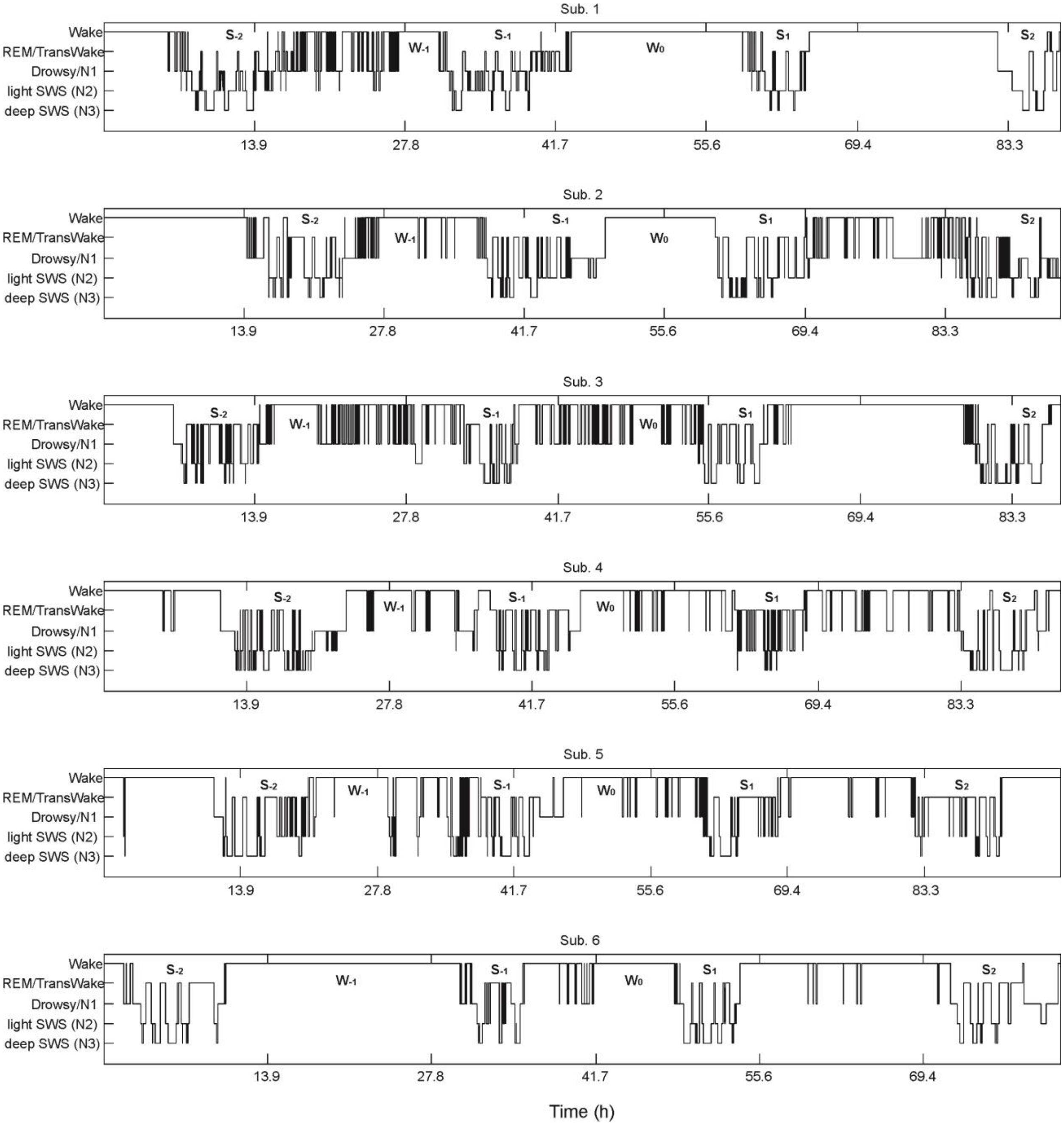

Supplementary Figure S2. Reconstructed hypnograms over four nights based on LFP characteristics resemble normal sleep structure. The separation of NREM and waking was achieved with the same methods used for Supplementary Fig. S3c. Time periods (< 2h) within the waking cluster of the PC state space that followed deep SWS (N3) periods and contain no

NREM sleep graphoelements were marked as REM/TransWake: REM or transitory waking period.

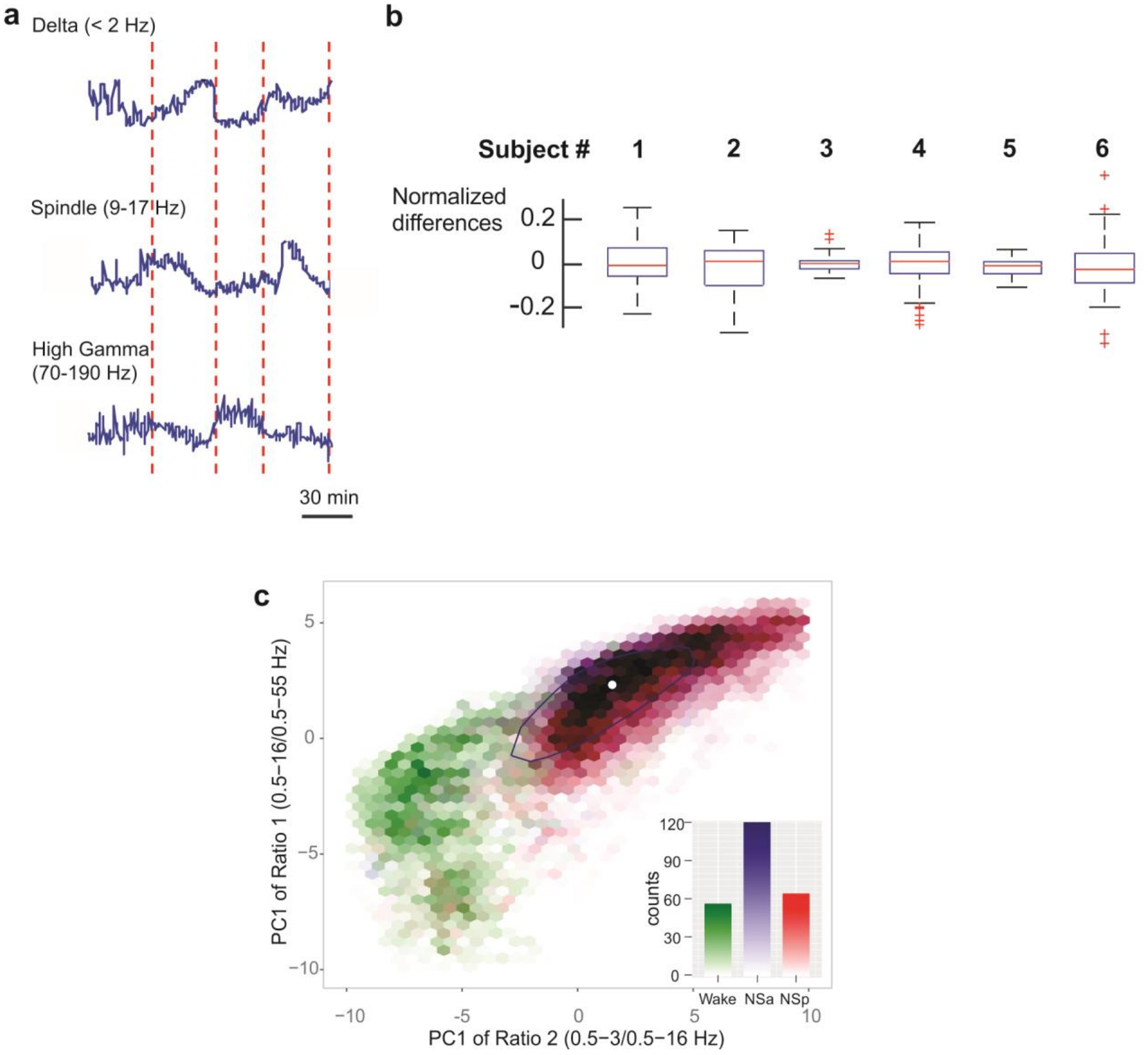

Supplementary Figure S3. **Selection of NREM periods**. ***a***, Identification of NREM sleep (stages N2+N3) using spectral power. The Hilbert analytic amplitudes of sleep LFP in the spindle (9-17 Hz), low delta (0.1-2 Hz), and HG (70-190 Hz) frequency bands were calculated, and the medians of consecutive 30s time windows were obtained. Prolonged delta amplitude

elevations in conjunction with drops in HG and generally high but gradually decreasing spindle frequency amplitude were used to identify NREM. ***b***, Similar depth of Sleep-Pre *versus* Sleep-Post NREM periods. The Normalized Difference was calculated from the channel-wise z-scored differences between Sleep-Pre and Sleep-Post delta amplitudes (sum of two Sleep-Pre NREM periods minus sum of two Sleep-Post NREM periods). For each subject, the blue box marks the range in Normalized Difference that covers the 25 % to 75 % percentile (interquartile range). The red lines mark the medians. The whiskers mark the maximum/minimum values except for outliers (+), i.e. channels with Normalized Difference values exceeding the blue box boundaries by 1.5 times the interquartile range. None of the subjects' medians were significantly different from 0 (one-sampled sign test, α=0.05). ***c***, NREM /wake separation via principal component analysis validates Hilbert amplitude-based NREM selection. Classifications by different methods of 2-second sleep epochs from the two subjects who were scored by polysomnographers are displayed as a 2D histogram. The first principal components (PCs) for two frequency ratios were computed across epochs and channels. Each hexagon represents all epochs with a particular value for PC1 of these ratios. RGB color blending for each hexagonal bin represents the proportion of sleep/wake epoch counts within the bin, with intensely single-colored bins representing frequently visited positions in state space. Lower right legend: NSa: epochs marked as NREM via Hilbert amplitude; NSp: epochs marked as NREM by qualified polysomnographers; Wake: epochs marked as waking by polysomnographers. There is substantial overlap (> 95 %) between NSa and NSp in PC space. The elliptic contour contains 50 % of all blue (NSa) epochs for each histogram, the white dot being the location of 2d median for blue epochs.

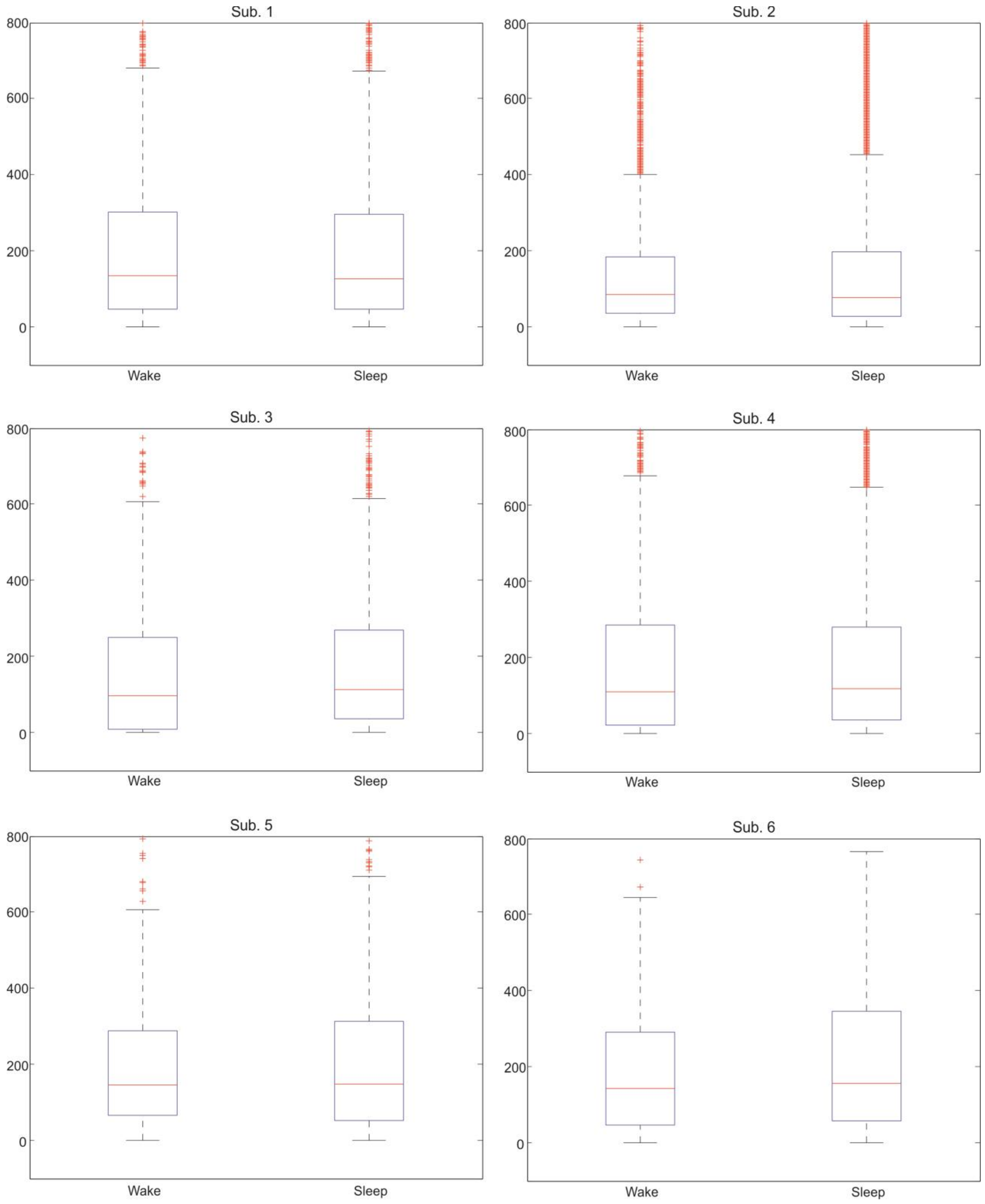

Supplementary Figure S4. Similar distribution of inter-peak intervals (IPIs, in milliseconds) within Events during waking and NREM. For each subject, the blue boxes mark

the ranges in waking or sleep Event IPIs that covers the 25 % to 75 % percentile (interquartile range). The red lines mark the medians. The whiskers mark the maximum/minimum values except for outliers (+), i.e. IPIs exceeding the blue box boundaries by 1.5 times the interquartile range. None of the subjects' waking Event IPI medians were significantly greater than the NREM Event IPI medians (Wilcoxon rank sum test, $\alpha = 0.05$).

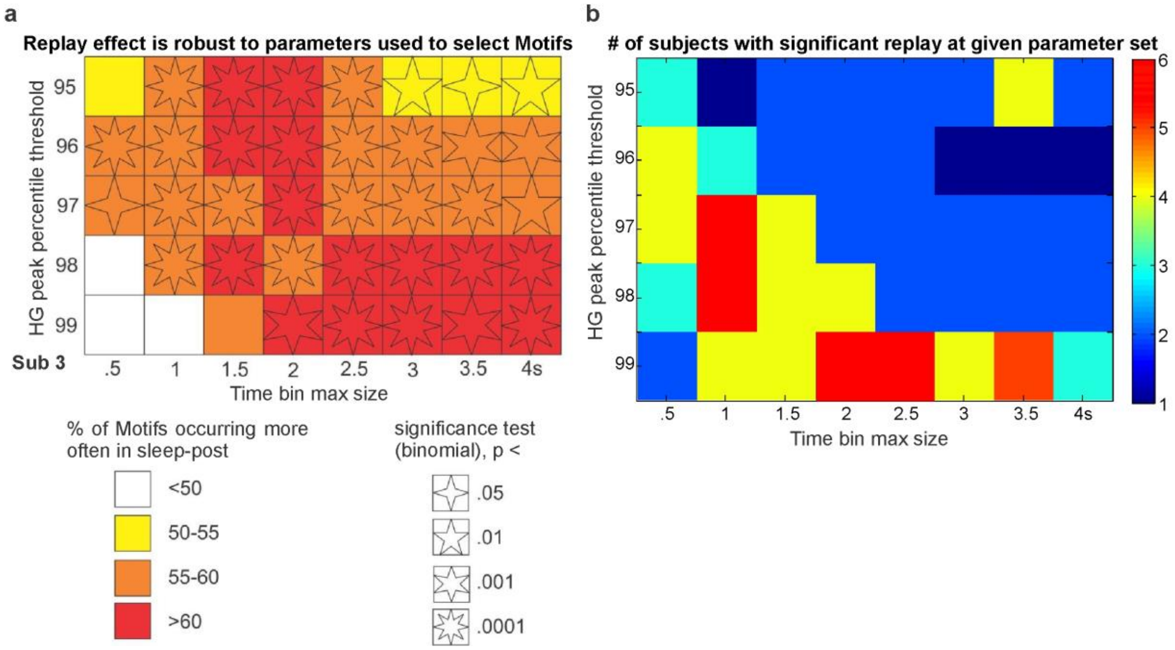

Supplementary Figure S5. Summary of significant replay observed under different HG peak thresholds and different maximum Event sizes. ***a***, Example summary of significant replay from subject 3. An identified waking Motif was considered replaying if it had more matching Events in Sleep-Post than in Sleep-Pre; the "replay effect" previously observed with HG peak threshold at 99th percentile and maximum Event size at 2 seconds was that the majority of waking Motifs were replaying. This effect is generally robust to changes in two crucial parameters used to select Motifs: HG peak threshold (from 95th to 99th percentile, in 1 percentile increments) and maximum Event size (from 0.5 s to 4 s, in 0.5 s increments). ***b***, Summary of the number of subjects, ranging from 1 to 6, showing a significant replay effect at each parameter set. All subjects showed significant replay effect at the following parameter sets ([HG percentile, Event size]): [97th, 1 s], [98th, 1 s], [99th, 2 s], [99th, 2.5s]. Note that, out of the 36 replay analyses with 99th percentile threshold and max Event sizes from 1 s to 3.5 s (6 parameter sets, 6 subjects), 29 showed significant replay effect, and 0 showed significance in the opposite direction (i.e. majority of waking Motifs matching Sleep-Pre Events more often than Sleep-Post

Events). Similarly, out of the 24 analyses with max Event size 1 s and percentile thresholds from 96th to 99th, 20 showed significant replay effect, and 0 showed significance in the other direction.

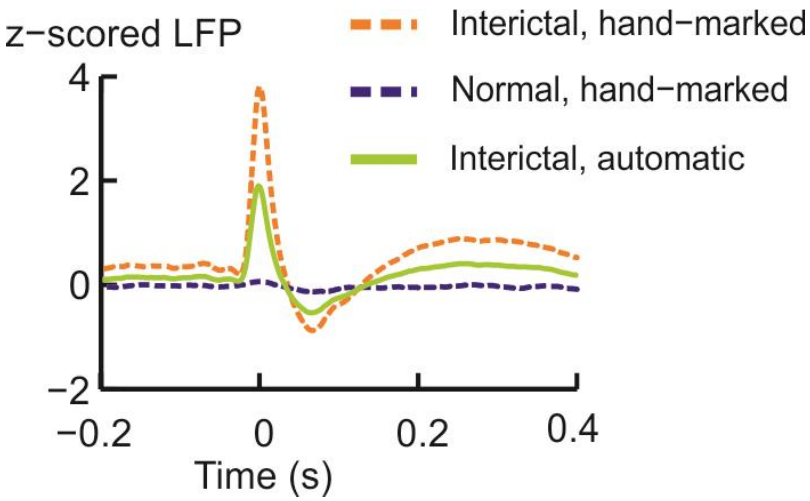

Supplementary Figure S6. Epileptic artifact rejection. ***b***, Artifact rejection via wavelet decomposition is sensitive and accurate. Out of a population of 990 2-second epochs (equal amounts of expert hand-marked interictal events/non-interictal random segments) taken from 3 channels of patient 2 with 0.1 Hz interictal occurrence rate, the artifact detector was able to capture most of the interictal events (53 false positives and 2 false negatives; d'=4.49). Consequently, the hand-marked and automatically-detected average waveforms are highly similar.

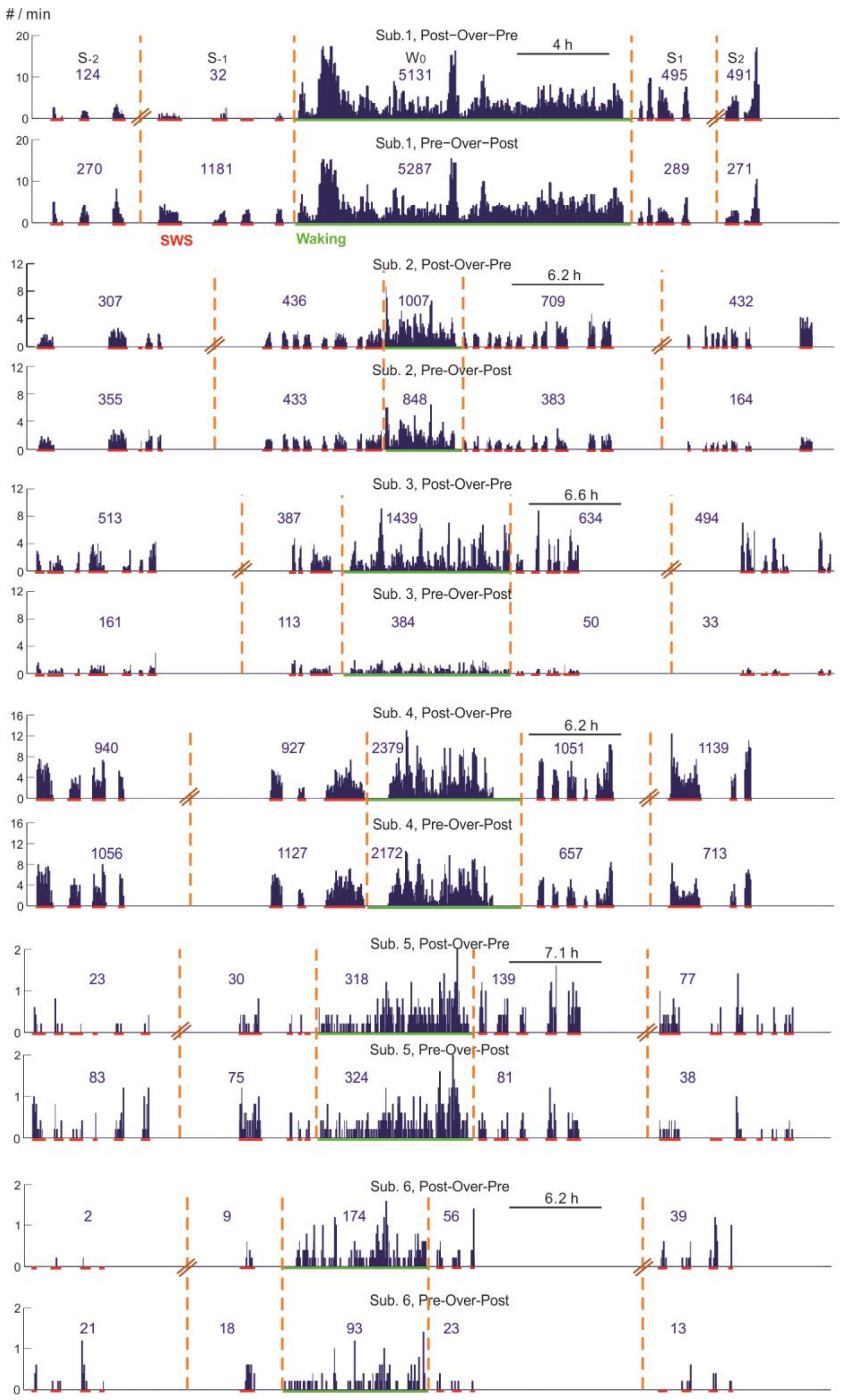

Supplementary Figure S7. The occurrence of significant (based on the combinatorial matching index) Motif-to-Event matches across four nights (S) and one waking period (W) to waking Motifs. NREM sleep (stages N2 and N3) segments are marked in red; waking

segments in green. Every two panels linked by orange dash lines belong to the same subject, with the diagonal double lines indicating separation between time periods. Histogram bin size: 5 min. Post-over-Pre: only showing Event matches to Motifs that matched more often to Events in Sleep-Post than to Events in Sleep-Pre. Pre-over-Post: only showing Event matches to Motifs that matched more often to Events in Sleep-Pre than to Events in Sleep-Post. The number of Event matches for each wake/sleep period is marked in blue above the corresponding period.

Subject ID	Replay analysis: Motif-Event matching (both exact and combinatiorial)	Replay analysis: Novel Motif-Event matching	Association of cortical graphoelements with Motifs	Association of hippocampal graphoelements with Motifs	Time-frequency analysis of hippocampal recording at Motif times	Enrichment of Motifs during task performance waking segments
1	Y	Y	Y	N	N	Y
2	Y	Y	Y	N	Y	Y
3	Y	Y	Y	Y	Y	N
4	Y	Y	Y	N	N	N
5	Y	N	Y	N	N	N
6	Y	N	Y	Y	Y	N
7	N	N	N	N	N	Y

Supplementary Table S1. List of subjects and the analyses performed with their electrophysiological recordings. Subject 7 was not used to perform the standard Motif-Event matching analyses since only one Sleep-Pre period was available (two would be required to perform replay analyses in the same manner as other subjects).

Supplementary Methods.

Waking Task Descriptions

The following tasks were performed by some of our subjects for other research groups, and we did not participate in the design or administration of these tasks. However, the electrophysiological recordings we received from our collaborators contained timing pulses, and we therefore were able to identify times of task participation during waking for three subjects.

NF task (subject 1): The Name-Face paired associate learning task consists of four consecutive phases: Nap1, Learning, Nap2, and Test, performed on the same day. At the Learning phase, the subject would learn to associate 3 unfamiliar name-face pairs. Some names would be presented also during Naps. No recordings from naps were analyzed in the current study.

AS task (subject 2): Subject heard a series of 7-10 s long tone sequences, each comprised of two pitches, and forming either a galloping rhythm or two parallel series, and the subject would report their experience.

Movie watching (subjects 1 and 7): Subjects 1 and 7 were provided with a laptop computer that contained movie content. Individual movie frames were aligned to trigger pulses sent to the clinical system, thus giving each movie frame a time stamp in electrophysiological data. Subject 1 watched a movie trailer lasting about 2.5 minutes four times, and was instructed to pay attention to different features each time before giving an oral report: 1. the characters, 2. the plot, 3. character/plot details not mentioned in previous oral reports, and 4. other details such as buildings and environmental sounds. Subject 7 was instructed to watch the movie “Zoolander” with no need for additional actions. Subject 7 completed viewing in one sitting, and did not

rewind or take breaks. The individual “Zoolander” movie frames were then extracted as jpeg images using the FFmpeg software, and all movie frames were loaded onto Google Picasa (version 3.9) to make use of its semiautomatic facial recognition feature (with 20 rounds of rescans and 20-40 hand marks per round), allowing each movie frame (and its associated electrophysiological data segment) to be marked as face-containing or not. Movie frames where both eyes were not visible on the same face were excluded from analysis to improve recognition accuracy.
